# MiR-129 blocks estrogen induction of NOTCH signaling activity in breast cancer stem-like cells

**DOI:** 10.18632/oncotarget.21143

**Published:** 2017-09-21

**Authors:** Guodong Xiao, Xiang Li, Gang Li, Boxiang Zhang, Chongwen Xu, Sida Qin, Ning Du, Jichang Wang, Shou-Ching Tang, Jing Zhang, Hong Ren, Ke Chen, Xin Sun

**Affiliations:** ^1^ Department of Thoracic Surgery and Oncology, The Second Department of Thoracic Surgery, Cancer Center, The First Affiliated Hospital of Xi’an Jiaotong University, Xi’an 710061, China; ^2^ Department of Otorhinolaryngology, The First Affiliated Hospital of Xi’an Jiaotong University, Xi’an 710061, China; ^3^ Department of Vascular and Endovascular Surgery, The First Affiliated Hospital of Xi'an Jiaotong University, Xi’an 710061, China; ^4^ Breast Cancer Program and Interdisciplinary Translational Research Team, Georgia Regents University Cancer Center, Augusta, Georgia 30912, United States; ^5^ Tianjin Medical University Cancer Institute and Hospital, Tianjin 300060, China; ^6^ Department of Urology, Union Hospital, Tongji Medical College, Huazhong University of Science and Technology, Wuhan 430022, China

**Keywords:** miR-129, ESR1, DICER1, Let-7, NOTCH signaling

## Abstract

Stem-like cells in tumor group featured the major role in the chemotherapy resistance of breast cancer, and the reduction of stem-like cells helped to perish the tumor when receiving chemotherapy. Smaller stem cells number indicated better therapeutic effect *in vitro* and in clinics, but how did miR-129 and Notch signaling function in breast cancer stem-like cells (BrCSCs) were unclear yet. Through using sphere forming assay and FACS sorting, we found that miR-129 decreased the proportion of stem-like cells in breast cancer cells. Results further indicated that miR-129 degraded the Estrogen Receptor 1 (ESR1) mRNA through a post-translational manner and contributed to the decline of stem-like cells number, preventing tumor regeneration. Cyclin d1 and DICER 1 were proved to promote Let-7 maturation, and in present study, we proved that miR-129 exhibited inhibition on ESR1 and halted the cyclin d1/DICER 1 sustaining of Let-7, which consequently released the Let-7 degradation of NUMB. The restoration of suppressive NUMB by upregulating miR-129 resulted in NOTCH signaling inhibition. In conclusion, we demonstrated the negative regulation of miR-129 on NOTCH signaling activation in BrCSCs’ renewal, which was achieved via continuous suppression on cyclin d1/DICER1 sustaining of Let-7 level, and eventually rescued the targeted inhibition of NUMB. The miR-129/ESR1 signaling played pivotal role in controlling DICER1/Let-7/NOTCH cascade via cyclin d1, revealing the novel mechanism of dual Let-7 in non-coding genes network.

## INTRODUCTION

Breast cancer is the most common malignancy among women in Europe and North America [1], and in developing countries, huge economic growth and accompanied dietary habit shift greatly contributed to growing incidence rate of breast cancer [2]. Despite the considerable progress in breast cancer treatment, metastasis and recurrence remain greatest challenges to clinicians. Growing evidences have shown that a sub-population of tumor cells, being named of cancer stem-like cells (CSCs), were responsible for cancer emergence, therapy resistance and long term recurrence [3]. These CSCs are endowed with stem cells’ signatures, playing critical role in cancer development and progression, and therefore became the final but toughest obstacle in battles for eliminating cancer [4–6]. Breast cancer stem-like cells (BrCSCs) are identified with cell surface phenotypes of either CD44^+^/CD24^*−*^ or aldehyde dehydrogenase 1 (ALDH1) [7–10], and it is now widely accepted that BrCSCs are functionally essential for tumor formation, progression, and therapy resistance in breast cancer patients [3, 11]. Thus, effective treatments targeting at BrCSCs may provide a novel strategy to improve patient outcomes.

In past few years, non-coding microRNAs (miRNAs) attracted much attention for their roles in cancer pathogenesis and progression [3, 12, 13]. MiR-129-5p, transcribed from miR-129-1 and miR-129-2, is one of the well-studied miRNAs associated with its tumor suppressive characteristics [14, 15]. Recent studies have reported that lower miR-129-5p expression in non-small cell lung cancer, breast cancer, hepatocellular carcinoma, ovarian cancer and colorectal cancer tissues, functioning through repressing cancer cell proliferation, invasion and multi-drug resistance in various cancers. MiR-129-5p reversed the epithelial-mesenchymal transition (EMT) process of breast cancer cells via direct regulations of twist1-snail feedback loop. However, little is known about the role of miR-129-5p in affecting the self-renewal ability of BrCSCs.

NOTCH signaling is activated by cell-to-cell interaction: NOTCH receptor (NOTCH 1, 2, 3 and 4) contact with ligands (Delta or Jagged proteins), prompting the release of NOTCH intracellular domain (NICD, Cleaved NOTCH1, activated NOTCH1), which then subsequently diffuses into the nucleus and interact with the co-activators to activate downstream events [16–20], and also contributed to the sphere formation ability of breast cancer stem cells [21, 22]. NUMB, a negative regulator of NOTCH signaling can also inhibit the NOTCH 1 by binding with Itch to degrade NOTCH receptors. In addition, NUMB interferes with the nuclear translocation of NICD through direct binding to it. The latest research showed that MAP17 (a Cargo Protein) induces an increase in stem cell factors and cancer-initiating–like cells by sequestering NUMB to activate NOTCH pathway [23]. One study recently identified that miR-129 could induce autophagic flux by targeted suppressing NOTCH-1 in glioma cells [18]. Furthermore, aberrant activation of the NOTCH signaling is associated with the stemness of BrCSCs [24–26]. These studies suggest that miRNA and NOTCH signaling axle may be the key regulatory mechanism in controlling breast cancer stem-like cells’ signatures.

Through informatics analysis, ESR1 and NUMB were predicated to be the potential downstream gene of miR-129 and Let-7 respectively. Based on these studies, we hypothesized that miR-129-5p might be a key regulatory factor of BrCSCs’ signatures through NUMB/NOTCH signaling axle. In the present study, we aimed to explore the miR-129 controlling of suppressive Let-7 in regulation of NUMB/NOTCH signaling, which will help to reveal the mechanistic involving of stem cells’ signature, paving the way for prospect strategy of eliminating cancer stem-like cells.

## RESULTS

### The proportion of stem cells harboring in cancer tissues was correlated to cancer stages

**Figure 1:**
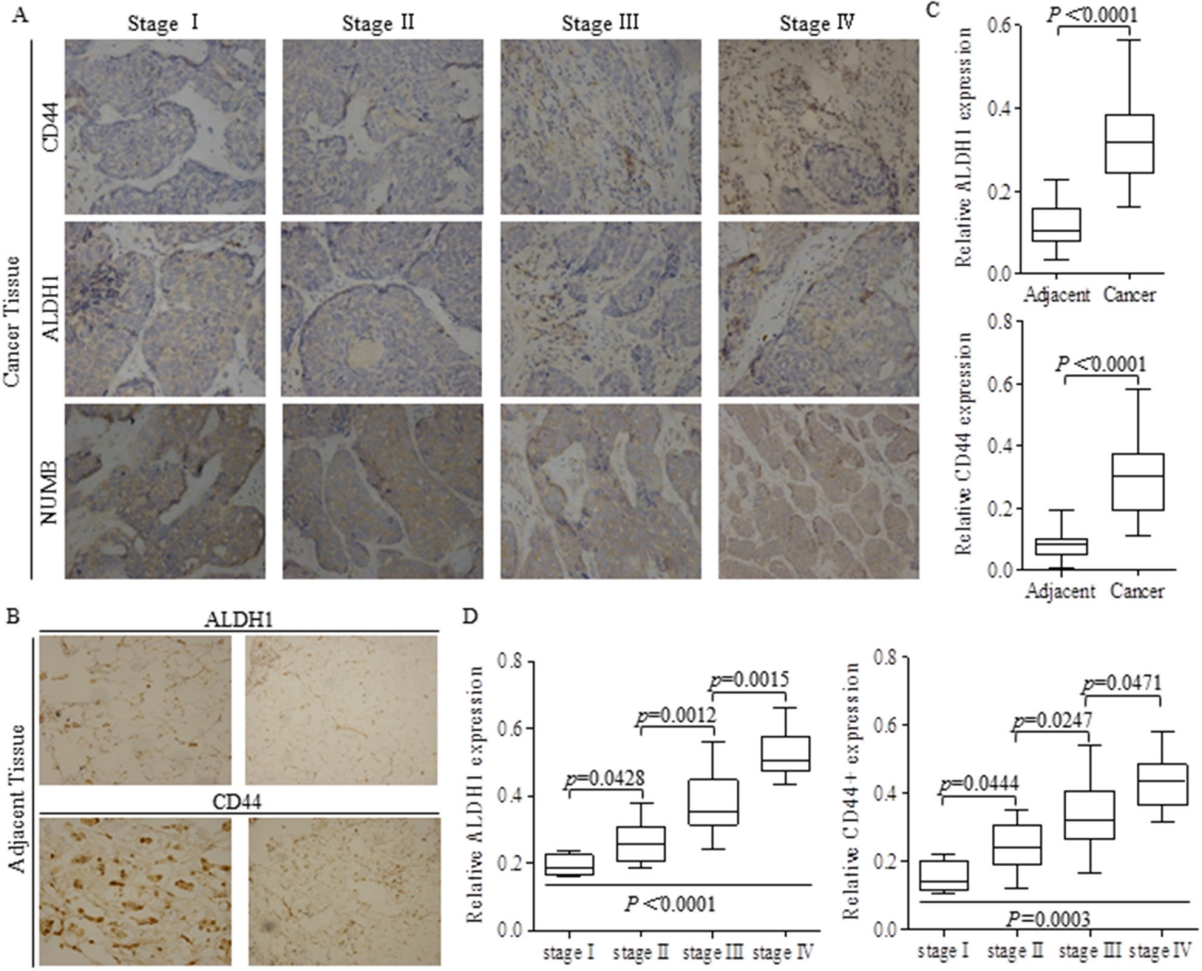
Higher proportion of stem-like cells stands for advanced carcinoma stages **(A)** Tissue slices of 42 from breast cancer patients of different stages were stained with ALDH1 or CD44 antibody respectively to detect the potential stem cells. **(B)** Adjacent tissues from patients with breast cancer were also stained with ALDH1 or CD44 antibody. **(C)** Relative intensities of ALDH1 and CD44 were both significant lower in adjacent tissues than those of cancer tissues. **(D)** Stem cells of ALDH1 or CD44 phenotype were higher in advanced carcinoma, and the p values were indicated in every specific location.

Forty two pairs breast cancer and matched adjacent tissues were stained with ALDH1 or CD44 antibody in each test, to detect the potential stem cells (Figure 1A-1B), and results showing that the intensities of either ALDH1 or CD44 was much stronger in cancer tissues than that of adjacent tissues (Figure 1B-1C). Stem-like cells of ALDH1 or CD44 positive phenotype featured to be enriched in patients with progressed breast cancer, indicating that the greater proportion of stem cells was correlated to advanced tumor stages (Figure 1D).

### The proportion of stem cells was inversely correlated with expression level of miR-129 and NUMB in clinical samples

**Figure 2:**
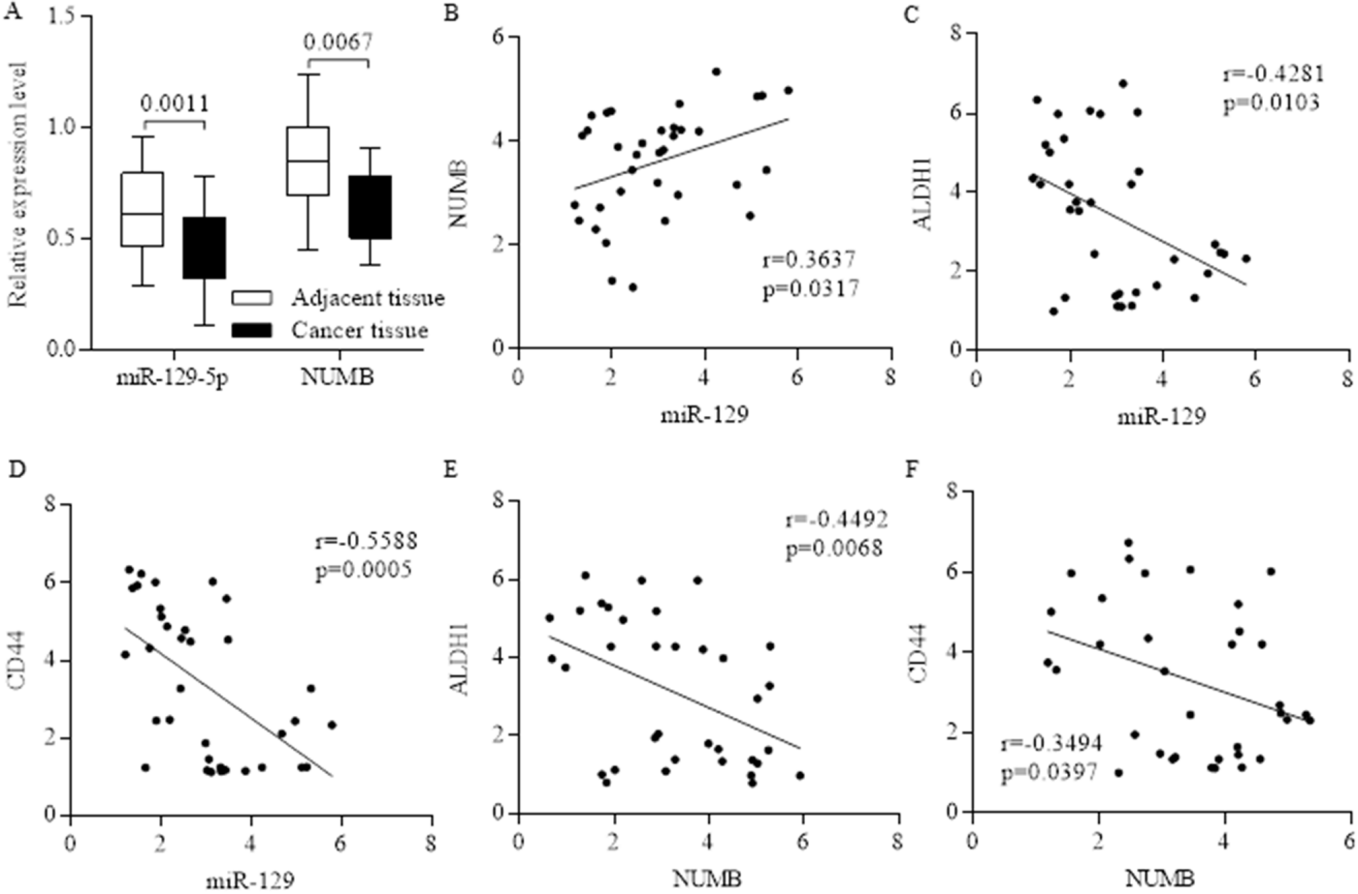
The inverse correlation between the proportion of stem cells and the expression level of miR-129 and NUMB Both miR-129 and NUMB were inhibited in breast cancer **(A)**, and we further identified the positive correlation between non-coding miR-129 and NUMB **(B)**. The expression level of miR-129 was inversely correlated to the stem cell’s marker of ALDH1**(C)** and CD44 **(D)**, and the correlation is significant. The expression level of NUMB was inversely correlated to the stem cell’s marker of ALDH1 **(E)** and CD44 **(F)**, indicating the potential suppressive roles of either miR-129 or NUMB in stem cells’ renewal.

Stem cell fate determination of NUMB is responsible for pluripotency and differentiation controlling, and was often suppressed in advanced carcinoma (Figure 1A, Figure 2A). MiR-129 was often inhibited in breast cancer, and further detection identified the positive correlation between non-coding miR-129 (Relative intensity) and NUMB (Figure 2B), which were both inversely corresponding to higher proportions of either ALDH1 or CD44 (Figure 2B-2F), indicating the potential suppressive roles of either miR-129 or NUMB in stem cells’ renewal.

### The decreased miR-129 in stem-like cells correlates directly with activation of ESR1 and NOTCH

**Figure 3:**
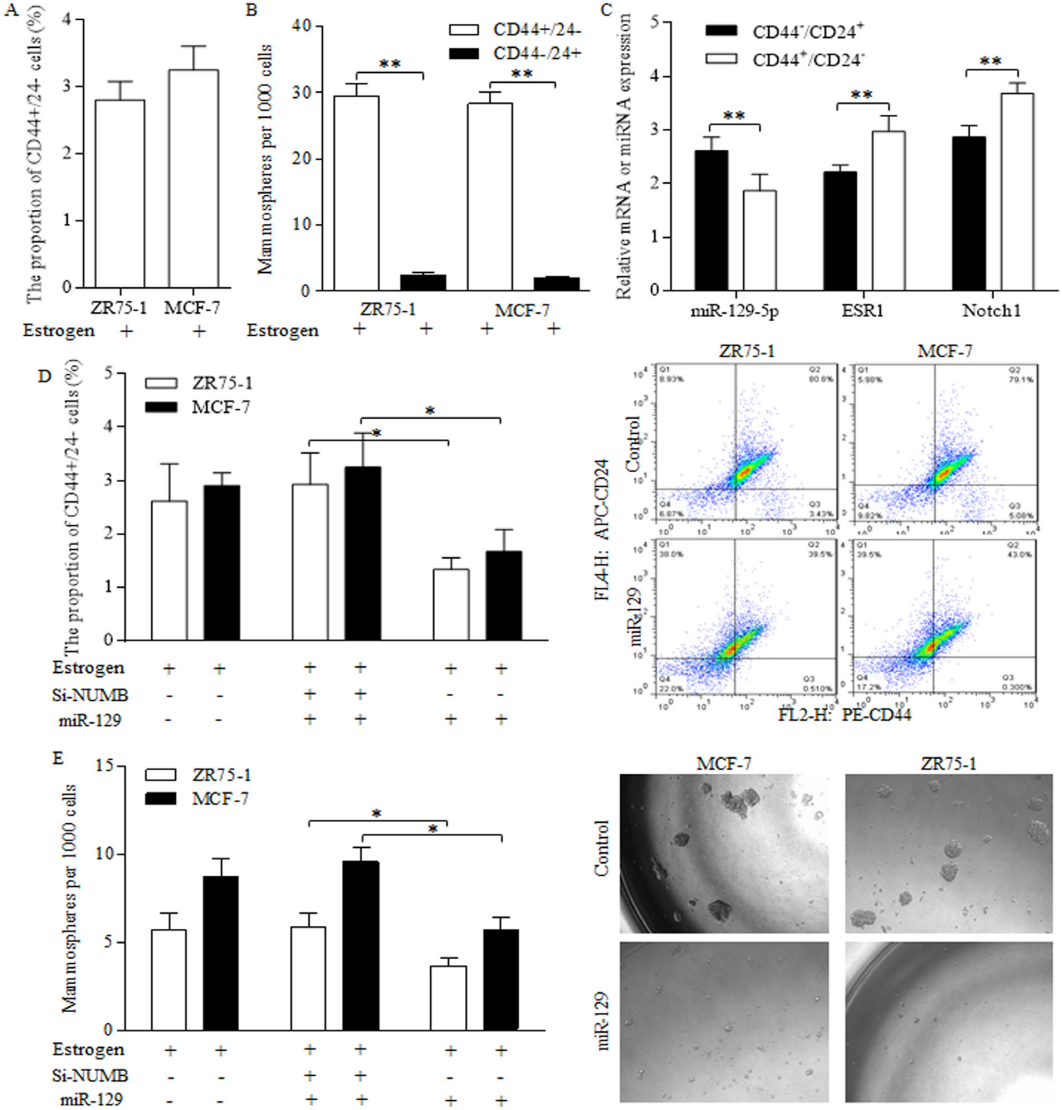
Suppressive miR-129 functions through inhibition on ESR1 and NOTCH signaling in breast cancer stem cells Breast cancer stem-like cells could be identified by CD44+/24- phenotype **(A)**, which were capable of forming much more spheres than the subtype of CD44-/24+ marker **(B)**. **(C)** MCF-7 stem-like cells of CD44+/24- phenotype preserved decreased miR-129 expression, together with upregulated ESR1 level and NOTCH 1, the NOTCH signaling activation indicator. **(D)** Enforced miR-129 expression decreased the proportion of CD44+/24- cells significantly, however NUMB inhibition induced NOTCH activation abolished the suppressive functions of miR-129. **(E)** MiR-129 decreased the mammosphere number, and the siRNA of NUMB induction of NOTCH activation abolished suppressive functions of miR-129, suggesting that miR-129 functioned through maintaining NUMB expression level.

Stem-like cells of CD44+/24- phenotype were identified in ZR75-1 and MCF-7 cells (Figure 3A), which possessed stronger ability of forming spheres than the subtype of CD44-/24+ marker (Figure 3B). In MCF-7 stem-like cells of CD44+/24- phenotype, we detected the decreased miR-129 expression, together with upregulated ESR1 level and NOTCH signaling indicator (Figure 3C). Enforced miR-129 expression decreased the proportion of CD44+/24- cells significantly (Figure 3D). Similarly, miR-129 decreased the mammosphere number acquired from suspended culturing (Figure 3E), which further consolidated the suppressive role of miR-129 in stem cells’ renewal. NUMB inhibition by siRNAs abolished suppressive functions of miR-129 (Figure 3D Left, Figure 3E Left), indicating the way miR-129 functioned was through maintaining NUMB expression level.

### Cancer stem-like cells were enriched through clinical neoadjuvant therapy

**Figure 4:**
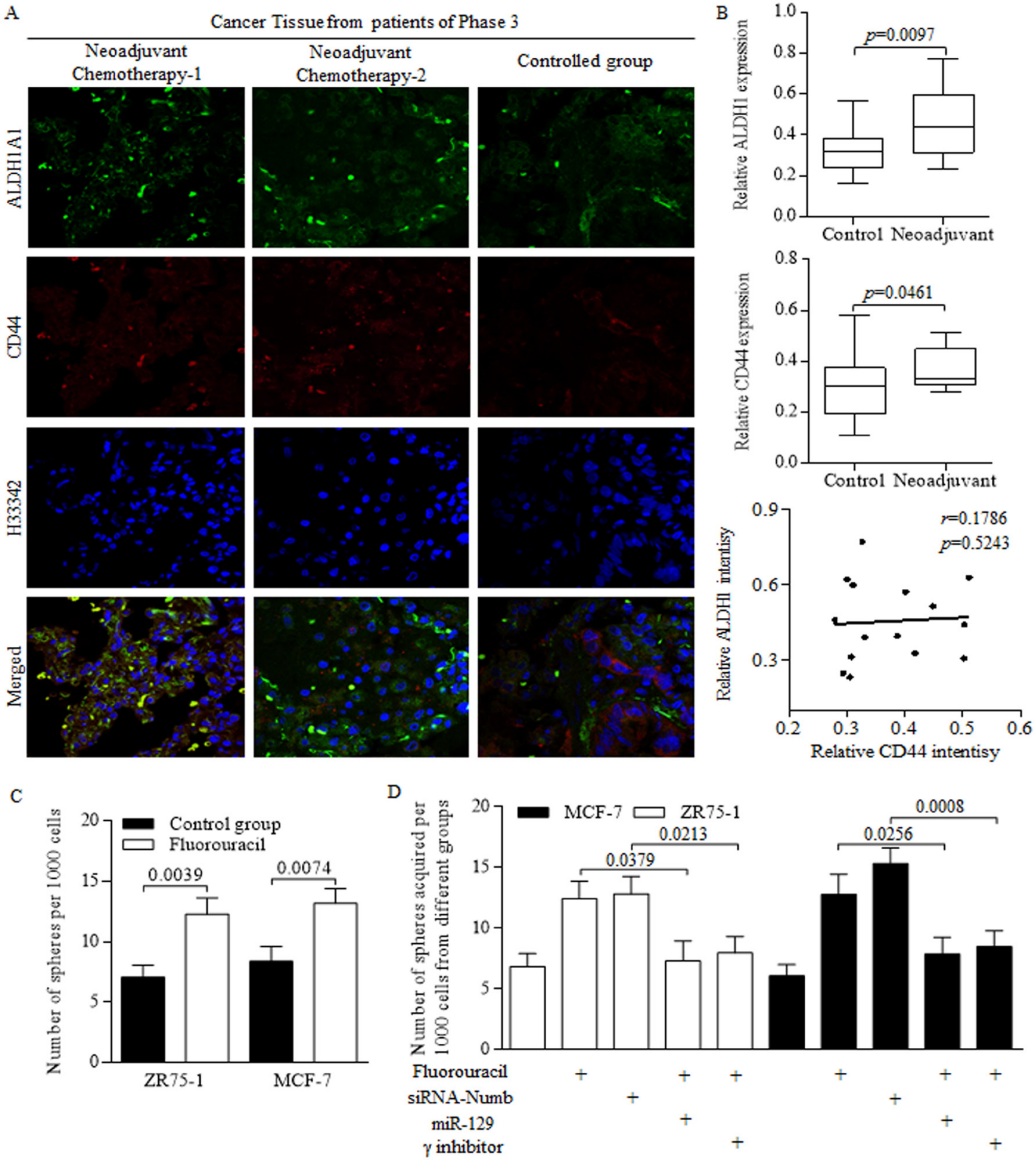
Chemotherapeutic treatments generates more cancer stem-like cells **(A)** Stem cells with CD44 or ALDH1 surface marker were awakened from hibernation after massive proliferative cancer cells being perished during the anticancer treatment. **(B)** Stem cells number became greater in patients received neoadjuvant chemotherapy, compared to that of patient received surgeries without neoadjuvant therapy, but no significant correlation was detected between ALDH1 and CD44 intensity. **(C)** Fluorouracil treatment increased the sphere formation efficiency of breast cancer stem-like cells. **(D)** Both Fluorouracil and NUMB inhibition facilitates the sphere forming ability, however, the enforced miR-129 and NICD inhibition significantly abolished the oncogenic promotion of Fluorouracil on self-renewing ability of cancer stem-like cells.

We previously identified the stem-like cells harboring in tumor group, which resulted in tumor regeneration and long term recurrence [3], however, with therapy related outcomes being barely explored and reviewed. The present study showed that the stem cells number became greater in patients received neoadjuvant chemotherapy, compared to that of patient received surgeries without neoadjuvant therapy (Figure 4A). In detail, the proportion of either ALDH1 positive or CD44 positive cells increased during chemotherapy process (Figure 4B). However, no significant correlation was detected between ALDH1 and CD44 intensity (Figure 4B). Further in studies of cancer stem-like cells, we identified the stimulation of chemotherapeutic agent of Fluorouracil on sphere formation efficiency (Figure 4C). The NUMB inhibition exerted similar facilitation as Fluorouracil did, however, the enforced miR-129 and NICD inhibition both abolished the oncogenic promotion of Fluorouracil on self-renewing ability of cancer stem-like cells (Figure 4D).

### MiR-129 suppressed Let-7b expression by inhibiting ESR1 directly

**Figure 5:**
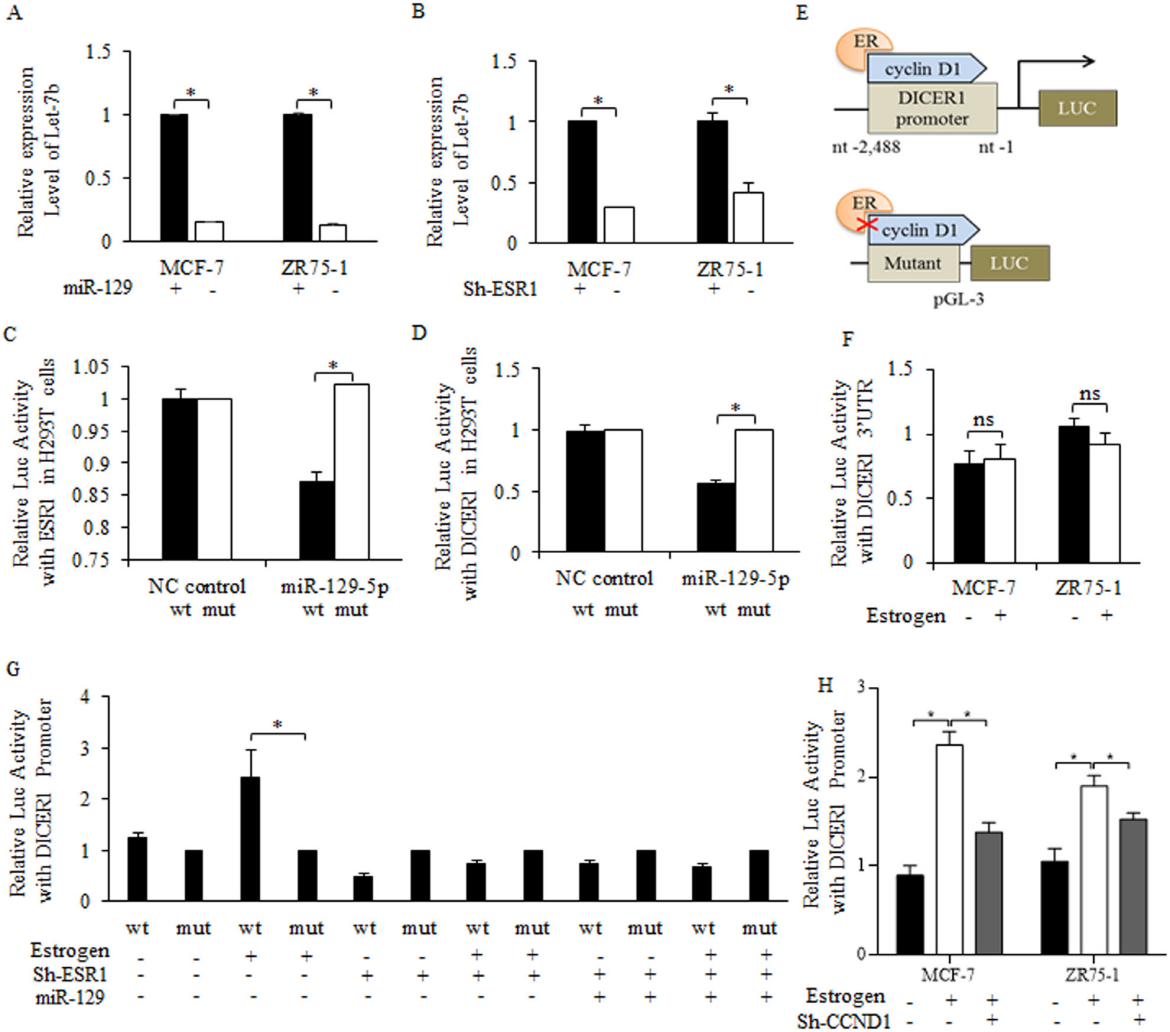
MiR-129 suppression of Let-7b level relied on direct inhibition on estrogen receptor sustained DICER1 activity Enforced miR-129 inhibited Let-7b expression level significantly **(A)**, so did the shRNA of estrogen receptor, and the results were significant **(B)**. **(C)** After introducing miR-129 mimic into 293T cells with wild type of ESR1 or mutant type of ESR1, we identified the direct binding site between the 3’UTR of wild type-ESR1 and miR-129, and miR-129 inhibited the ESR1 level significantly. Overexpressed miR-129 suppressed the DICER1 promoter activity significantly **(D)**. **(E)** The illustration of cyclin D1-ER regulated Dicer-pGL3 Luc-vector, estrogen treatment upregulated promoter activity of cyclin D1 sustained Dicer 1, and the both depletion or blocked of cyclin D1, and the mutant Dicer 1 promoter abolished the estrogen functions. **(F)** Little effects were detected on the luc-activity of DICER1 3’UTR when treating with estrogen (10 nM Estradiol) (Above), on the contrary, DICER1 promoter changed significantly (Below). **(G)** 10 nM Estradiol treatment stimulated the promoter activity of DICER1 promoter, but little effects were detected when either introducing miR-129 or inhibiting ESR1, and most importantly, no significant difference occurred between the individual regulation of miR-129 or ESR1, and the combined regulation. *p<0.05. **(H)** Estrogen stimulation of DICER1 activity was dependent on cyclin d1, and the inhibition of cyclin d1 abolished the activation of estrogen on DICER1 promoter.

To uncover the mechanistic connection between miR-129 and Let-7b, we first enriched and isolated breast cancer stem cells by mammosphere culture, and then introduced miR-129 mimic into breast cancer stem-like cells. MiR-129 overexpression effectively inhibited Let-7b level (Figure 5A), so did the shRNA of estrogen receptor (Figure 5B). Potential binding sites between miR-129 and the 3’UTR of ESR1 was detected after analyzing and searching results of DIANA-MICROT, MIRDB, MICRORNA.ORG, TARGETSCAN-VERT, TARGETSCAN-VERT online (data not shown). Bioinformatics analysis of “Target Scan” was recorded and filed (Supplementary Figure 1A). By introducing miR-129 mimic into 293T cells with ESR1-WT or ESR1-MUT (Supplementary Figure 1B), we identified the direct binding site between the 3’UTR of wild type-ESR1 and miR-129 (Figure 5C).

### Let-7b decreasing was attributed to DICER1 suppression

DICER1 enzyme controlled miRNAs maturation, and its stability could be sustained with the existence of cyclin d1 [27]. On the premise that estrogen interacted with cyclin d1 in multiple levels, we hypothesized that miR-129 regulation of Let-7b may be achieved through an ESR1/cyclin d1/DICER1 signaling cascade. By using Luc-plasmid built DICER1 promoter, we found the decreased activity of DICER1 correlates with miR-129 overexpression (Figure 5D), and the scheme was illustrated in Figure 5E. Further results of luciferase assay consolidated the theory that DICER1 activity was controlled indirectly through ESR1/cyclin d1/DICER1 signaling, as the 3’UTR of DICER1 did not change significantly when introducing miR-129 (Figure 5F). Estrogen-stimulation of DICER1 activity was neutralized when increasing miR-129 level or inhibiting estrogen receptor expression in cancer stem-like cells (Figure 5G). Co-regulation of miR-129 and ESR1 did not reduce the DICER1 activity further (Figure 5G), suggesting miR-129 regulation of DICER1 was dependent on the existence of ESR1 (Figure 5G). Estrogen treatment stimulated DICER1 promoter activity dependent on cyclin d1 existence (Figure 5H).

### MiR-129 inhibited NOTCH signaling activity through a Let-7b deficiency dependent manner

**Figure 6:**
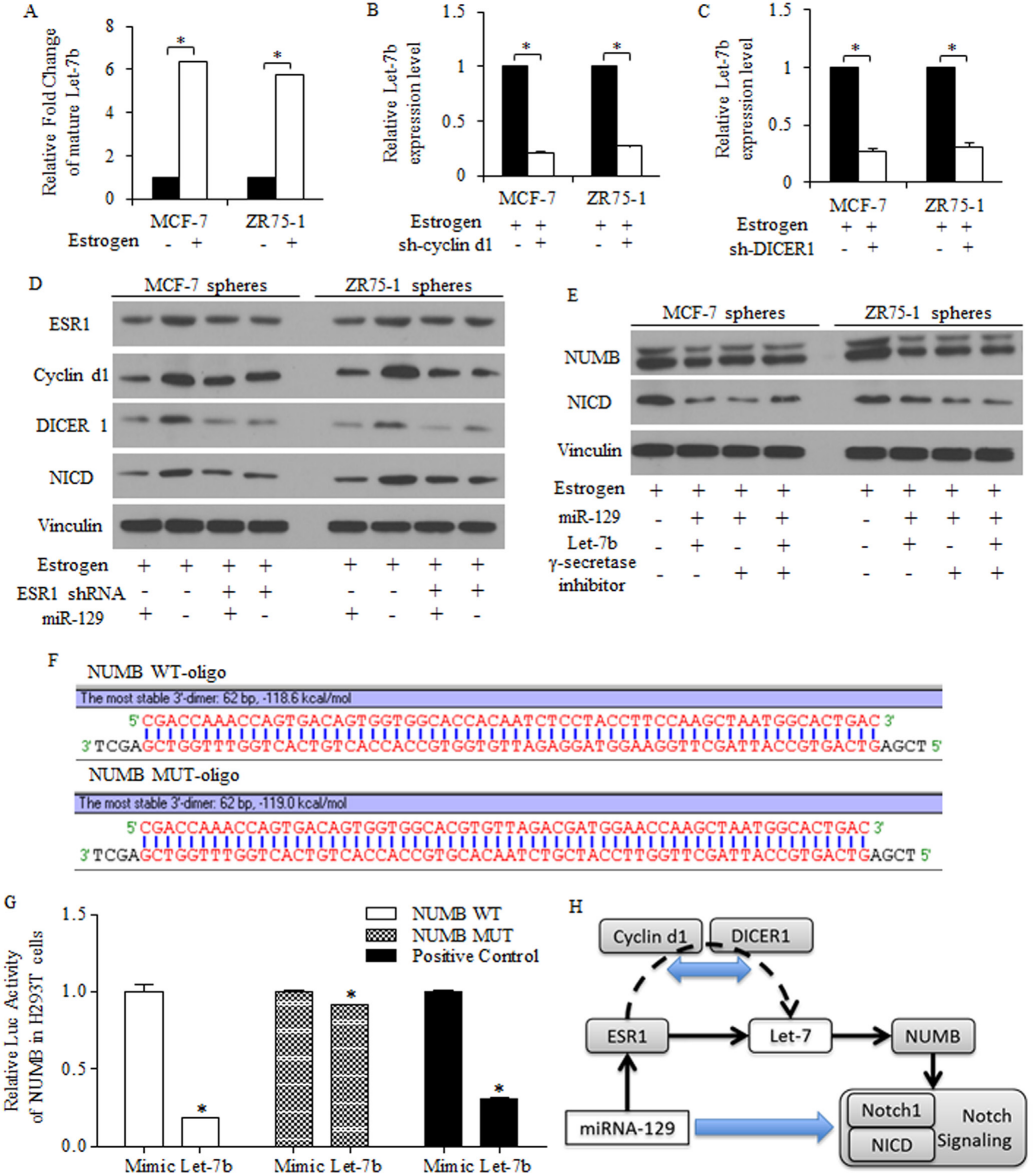
MiR-129 deprived the Let-7 inhibition on NUMB expression and functioned through NOTCH signaling depression **(A)** 10 nM Estradiol treatment increased Let-7b expression level in breast cancer cells. Let-7b decreased due to the absence of cyclin d1 **(B)** or DICER1 **(C)**. **(D)** MiR-129 functioned through inhibiting ESR1/cyclin d1/NOTCH pathway in breast cancer stem-like cells of spheres, and overexpressed miR-129 decreased ESR1, cyclin d1 and NICD, detected by immunoblotting. MiR-129 inhibition on DICER1 contributed to Let-7b inhibition (C), and overexpressed miR-129 inhibited NICD through inhibiting Let-7b, and no significant difference was identified between the second and the third row **(E)**. **(F)** Let-7b degraded NUMB expression through a 3’UTR binding way, and the scheme of 3’UTR regions were drafted. **(G)** Let-7b decreased the luc-activity of NUMB 3’UTR significantly. **(H)** Let-7b expression could be attenuated by miR-129, contributing to the suppressive roles of miR-129, and the inhibition of BrCSCs was achieved through the ESR1/Let7b/NUMB signaling, in the presence of cyclin d1 and DICER1.

To study the mechanisms of Let-7 dysregulation in breast cancer stem-like cells, we found estrogen treatment increased Let-7b expression level (Figure 6A), which could be reversed by either cyclin d1 knockdown (Figure 6B) or DICER1 knockdown (Figure 6C). Together with results acquired above, we hypothesized that the suppressive roles of miR-129 may be attributed to the active pathway of miR-129/ESR1/Let-7b in groups of stem-like cells in breast cancer, dependent on the existence of cyclin d1/DICER1.

Immunoblotting results identifying the suppression of miR-129 exerted on ESR1 and its downstream cyclin d1 in breast cancer stem-like cells (Figure 6D). By pasting the list of potential targeted genes of miR-129 into KEGG pathway analysis site, we noticed the pathways miR-129 may participate, especially for stemness signatures regulation (data not shown). MiR-129 inhibits NOTCH signaling through NICD inhibition, as γ-secretase inhibitor did, and restored Let-7b alleviated miR-129 resulted NUMB increasing, suggesting that Let-7 deletion is required for miR-129 functions in a miR-129/ESR1/NOTCH manner, but not sufficient for stem cells suppression (Figure 6E).

Let-7b degraded NUMB expression through a 3’UTR binding way (Figure 6F-6G), and Let-7b could be attenuated by miR-129, suggesting the Let-7b suppression contributed to suppressive roles of miR-129 (Figure 6G). The activation of key factors of NOTCH signaling pathway was correlated to stem-like cells harboring and long term tumor recurrence, and rescued NUMB by miR-129 inhibited NICD (Figure 6G).

### Stem-like cells were prevented from forming tumor *in vivo*

**Figure 7:**
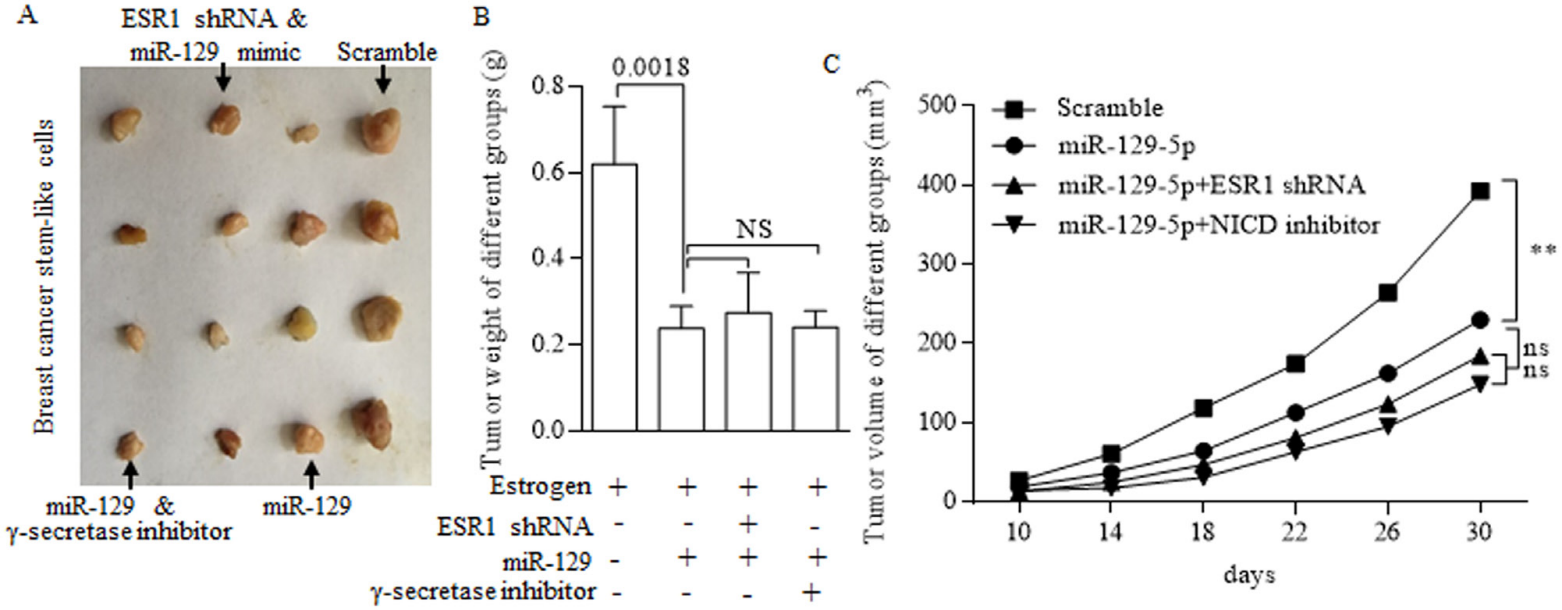
*In vivo* study of suppressive miR-129 **(A)** Tumors acquired from subcutaneously implanted estrogen-treated MCF-7 stem cells of different groups. Overexpressed miR-129 inhibits both tumor weight **(B)** and tumor growth **(C).** When combined with either ESR1 inhibition or γ-secretase inhibitor, no significant difference was drawn from signal use and combination of tumor weight (B) and tumor growth (C).

To investigate the role of miR-129 in inhibiting the growth of stem-like cells *in vivo*, we subcutaneously implanted estrogen-treated MCF-7 stem cells that stably overexpressed miR-129 into immune deficient mice to form tumors. Tumor size was measured every five days, when the last measurement was performed (Figure 7A). Results showed that overexpressed miR-129 could inhibit both tumor growth and tumor weight (Figure 7B-7C).

## DISCUSSION

Stemness controlling factors of Wnt, NOTCH, HH signaling, TGF-β signaling, and other pluripotency stimulators [28–30] were accused for sustaining the group of cancer stem cells in specific niche [31, 32], being accused for expanding the cancer clan. The targeted therapy focusing on cancer stem cells has been hot spot for year, which may help to reveal the prospect future of eliminating carcinoma entirely [33, 34]. Roles of non-coding genes in regulations of cancer biology were extensively studied, and the intrinsic interactions among multiple non-coding genes attracted more attention [35–37].

Cell fate determination of NUMB controlled the stem cells differentiation through its dominating action in suppressing NOTCH signaling [38, 39]. Many oncogenic miRNAs were proved to function through negative posttranscriptional regulation of NUMB [40–42], however, the way suppressive miRNA of Let-7 functioned through in regulating NUMB has not been covered.

In this study, we showed that the expression of miR-129 and NUMB were negatively correlated with the proportion of stem cells in tissues of breast cancer. Further, we proved that miR-129 executed inhibition on BrCSCs through directly targeting and degrading ESR1 expression, which could induce activation of the NOTCH signaling. Enforced miR-129 reduced the stem cells number of breast cancer, which was achieved by post-translational regulation of ESR1 level. Decreased cyclin d1 failed to support the DICER1 sustaining of mature Let-7, due to the initiation of ESR1 declining conducted by miR-129 suppression of ESR1. Consequently, the Let-7 decreasing released the targeted down-regulation of NUMB, contributing the NOTCH oncogenic signaling inhibition.

In conclusion, we demonstrated the complicated mode of miR-129 regulation of NOTCH signaling in stem cells’ renewal prevention, via continuous suppression on cyclin d1/DICER1 sustaining of Let-7 level, establishing the miR-129/ESR1/cyclin d1/DICER1/Let-7 regulatory axis. But most importantly, the dual roles of Let-7 in miR-129 dominated self-renewal inhibition were not sufficient to predicated its oncogenic characters, being involving in network always makes diverse changes.

## MATERIALS AND METHODS

### Cell culture, reagents and clinical specimens

Breast cancer cells of MCF-7 and ZR75-1 cell lines were purchased from ATCC. HEK293T was purchased from the Cell Bank of the Chinese Academy of Sciences. All cells were maintained in DMEM medium supplemented with 10% FBS (BI, Biological Industries), 1% Penicillin-Streptomycin Solution (Hyclone) at 37°C in a humidified incubator with 5% CO2. Cell were transferred into special medium (phenol red free) added with charcoal-dextran treated serum before estradiol treatment. For sphere formation assay, no serum was added, as was introduced below. MiR-129 mimics/inhibitors, Let-7b mimics/inhibitors, miR-129 lentiviral based RFP-plasmin were synthesized by GenePharma. Estradiol (estrogen, E1024 Sigma-Aldrich), 5-Fluorouracil (F6627S Sigma-Aldrich), γ-secretase inhibitor (SCP0004 Sigma-Aldrich) were purchased and stored in the central laboratory. Forty-two pairs clinical samples were collected from January 2011 to January 2015 (Supplementary Table 1), and written informed consents were obtained and filed. The medical ethics committee of the institution of Xi’an Jiaotong University approved this study.

### Quantitative reverse transcription PCR (qRT-PCR)

Total RNA from culture cells or clinical tissues was extracted with TRIzol Reagent (Invitrogen), and reverse transcribed into cDNA with TaqMan™ MicroRNA Reverse Transcription Kit (Thermo Scientific, #4366596). The relative quantification of miR-129 and Let-7b was performed using SYBR® Green PCR Master Mix (TaKaRa, Takara Code: 639676); GAPDH and U6 were used as endogenous controls. Primers for mature miR-129 and Let-7b were purchased from RiboBio. Each RNA sample was repeatedly detected in triplicate and statistically analyzed as previously did [13, 35].

### Lentiviral infection, transient transfection and plasmid construction

Cyclin D1 siRNA (human) (sc-40489, Santa Cruz Biotechnology), Dicer siRNA (human) (sc-40489, Santa Cruz Biotechnology) and ESR1 siRNA (human) (sc-29305, Santa Cruz Biotechnology) were used for Knocking down [13, 27]. Scrambled siRNA (sc-37007, Santa Cruz Biotechnology) was used as control. All small RNAs were transfected into MCF-7 or ZR75-1 cells by using Lipofectamine 3000 (Invitrogen) according to the manufacturer’s instructions.

To construct the luciferase expression vector, the 3’-UTR containing the predicted miR-129-5p binding sites (wild-type, WT) and mutated 3’-UTR (Mut) of the human DICER1 gene were obtained by PCR, and inserted into the pGL3-Control. The recombinant plasmid was named pGL3-DICER1-WT and pGL3-DICER1-Mut, as previously did [27, 35]. We also constructed the pGL3-ESR1-WT and pGL3-ESR1-Mut. The wildtype or mutant promoter regions of DICER1 was amplified and ligated into pGL3 reporter vector, and named as pGL3-DICER1p-Wt and pGL3-DICER1p-Mut. To identify the possible binding site between 3’-NUMB and Let-7b, a 62-bp fragment of NUMB 3’UTR was subcloned into the PmirGLO control vector to generate PmirGLO-NUMB-3’UTR-wt, Mutant construct of NUMB 3’UTR, named as PmirGLO-NUMB-3’UTR-mut, which carried a substitution of nucleotides in the core seed sequence of Let-7b.

### Western blotting and immunohistochemically staining

Western blotting and Immunohistochemically staining were performed as previously described [35]. The primary antibodies used in WB concludes of ESR1 (ab108398, 1:2000, Abcam), cyclin d1(#2978, 1:2000, Cell Signaling Technology), DICER1 (#3363, 1:2000, Cell Signaling Technology), NICD (#2421, 1:1000, Cell Signaling Technology), Vinculin (#13901, 1:5000, Cell Signaling Technology), NUMB (ab14140, 1:2000, Abcam), GADPH (#5174, 1:5000, Cell Signaling Technology). For immunobiological assay, the slides were incubated with anti-CD44 antibody (#3570, 1:500, Cell Signaling Technology), ALDH1 antibody (#54135, 1:500, Cell Signaling Technology) and NUMB antibody (ab14140, 1:1000, Abcam).

### Luciferase assays

HEK293T cells were seeded in 24-well plates and co-transfected with miR-129-5p mimics, miR-Ctrl and pGL3-ESR1-WT, pGL3-ESR1-Mut, pGL3-Dicer1-WT or pGL3-Dicer1-Mut. Luciferase activities were measured at 48h after transfection using a luciferase assay kit (Promega, Madison, USA). The promoter reporter plasmid pGL3-Dicer1p-WT or pGL3-Dicer1p-Mut was co-transfected with miR-129-5p mimic or ESR1shRNA or treated with estrogen (10 nM Estradiol) into HEK293T cells.

### Fluorescence activated cell-sorting (FACS) analysis

For analysis of BrCSC marker CD44 and CD24 expression, cells in different treatment groups were collected and washed with PBS twice. Then, the cells were incubated with anti-CD44-FITC (Sigma SAB4700189) and anti-CD24-PE (Sigma SAB4700625) antibodies at 4°C for 30 minutes. After washing two times, the samples were analyzed using a flow cytometer (FACScalibur, BD Biosciences).

### Mammosphere formation assay

Mammospheres were cultured under the condition as described previously, specifically, chemically defined cell culture Media, 3dGRO™ Spheroid Medium (S3077, Sigma-Aldrich) was applied, using Ultra-Low attachment plates (CLS3471, Corning) [13, 35]. After culturing for approximately 7 days, the number of the spheres/2000 cells were counted and scored under an inverted microscope. Sphere formation efficiency = colonies/input cells ×100%. Serial passage was achieved by enzymatic dissociation with Trypsin-EDTA (T4049, Sigma-Aldrich) and Accutase® solution (A6964, Sigma-Aldrich).

### Nude mice study

Spheres of MCF-7 cells were pretreated with estrogen (10 nM Estradiol), and then were disaggregated into single cell using combined reagents of trypsin (Cellgro, CORNING, USA) and Accutase (StemPro, GIBCO, USA). Stem-like cells were implanted subcutaneously into 5 weeks old nude mice supplied by the Experimental Animal Center, College of Medicine, Xi’an Jiaotong University (License No. SCXK (Shan) 2007-001) [4–7]. Long-release estrogen pellets (Innovative Research of America, USA) were implanted the day before inoculation. Tumor growth was measured by a digital caliper every 5 days for 5 weeks. Tumor weight was measured after cell implantation. Animal experimental researches followed the internationally recognized guidelines. Protocols were conducted in accordance with the Guidance for the Care and Use of Laboratory Animals, which established by the Ministry of Science and Technology of the People’s Republic of China.

### Statistical analysis

Data from experiments were statically analyzed using the SPSS statistics software version 16.0 and Prism 5.0. Quantitative data are presented as means±SD for at least three observations per group. Statistical differences among three or more groups are determined by using One-way ANOVA or Student t test. In all cases, P < 0.05 was considered statistically significant.

## SUPPLEMENTARY MATERIALS FIGURE AND TABLE


