# Correction: MiR-129 blocks estrogen induction of NOTCH signaling activity in breast cancer stem-like cells

**DOI:** 10.18632/oncotarget.28201

**Published:** 2023-09-15

**Authors:** Guodong Xiao, Xiang Li, Gang Li, Boxiang Zhang, Chongwen Xu, Sida Qin, Ning Du, Jichang Wang, Shou-Ching Tang, Jing Zhang, Hong Ren, Ke Chen, Xin Sun

**Affiliations:** ^1^Department of Thoracic Surgery and Oncology, The Second Department of Thoracic Surgery, Cancer Center, The First Affiliated Hospital of Xi’an Jiaotong University, Xi’an 710061, China; ^2^Department of Otorhinolaryngology, The First Affiliated Hospital of Xi’an Jiaotong University, Xi’an 710061, China; ^3^Department of Vascular and Endovascular Surgery, The First Affiliated Hospital of Xi’an Jiaotong University, Xi’an 710061, China; ^4^Breast Cancer Program and Interdisciplinary Translational Research Team, Georgia Regents University Cancer Center, Augusta, Georgia 30912, United States; ^5^Tianjin Medical University Cancer Institute and Hospital, Tianjin 300060, China; ^6^Department of Urology, Union Hospital, Tongji Medical College, Huazhong University of Science and Technology, Wuhan 430022, China; ^*^These authors are considered as co-first authors

**This article has been corrected:** In Figure 3D, the MCF-7 ‘Control’ image is an accidental duplicate of the ZR75-1 ‘Control’ image. The corrected Figure 3, produced using the original data, is shown below. The authors declare that these corrections do not change the results or conclusions of this paper.

Original article: Oncotarget. 2017; 8:103261–103273. 103261-103273. https://doi.org/10.18632/oncotarget.21143

**Figure 3:**
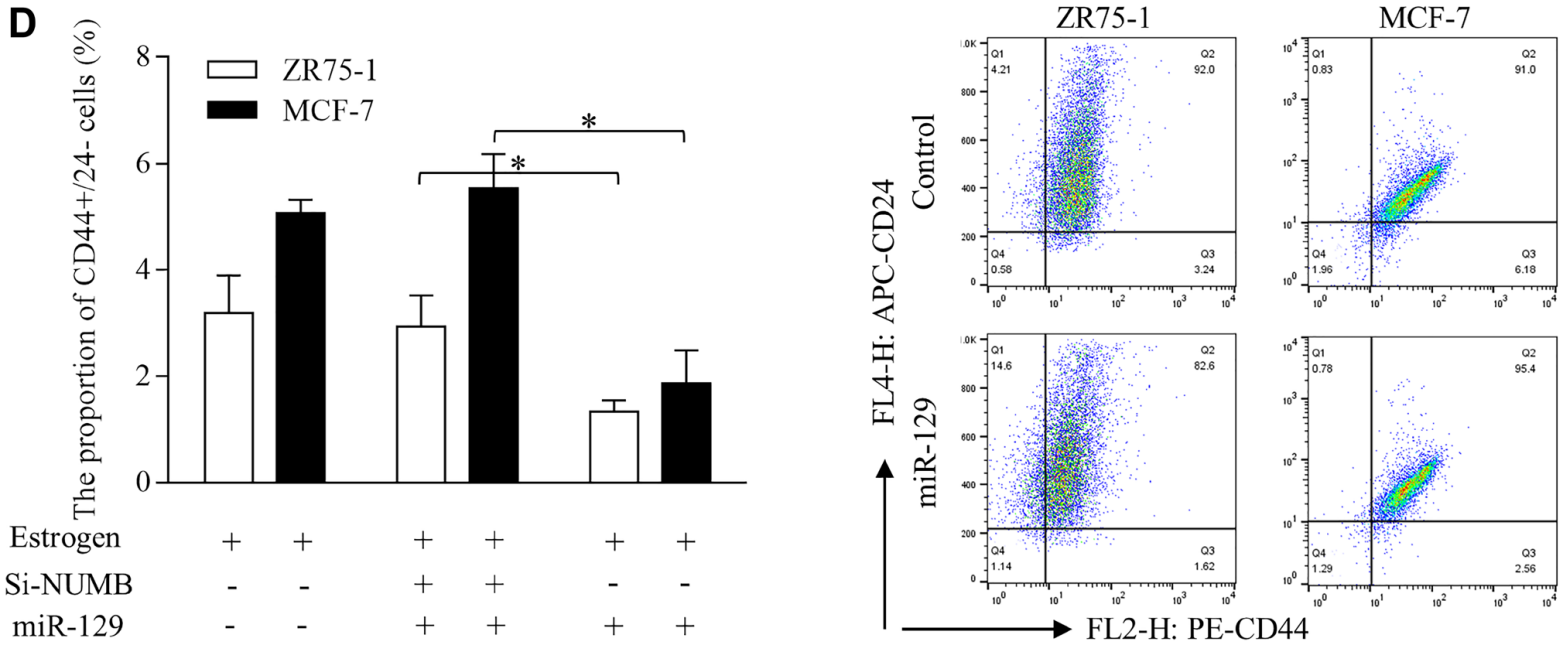
Suppressive miR-129 functions through inhibition on ESR1 and NOTCH signaling in breast cancer stem cells. (**D**) Enforced miR-129 expression decreased the proportion of CD44+/24− cells significantly, however NUMB inhibition induced NOTCH activation abolished the suppressive functions of miR-129.

