# Supplementary material for: MiR-129 blocks estrogen induction of NOTCH signaling activity in breast cancer stem-like cells
Source: Oncotarget. 2017 Sep 21;8(61):103261–73. doi: 10.18632/oncotarget.21143 (PMC5732725; doi:10.18632/oncotarget.21143)
Supplement: Supplementary file 1 [file oncotarget-08-103261-s001.pdf]

MiR-129 blocks estrogen induction of NOTCH signaling activity in breast cancer stem-like cells

SUPPLEMENTARY MATERIALS

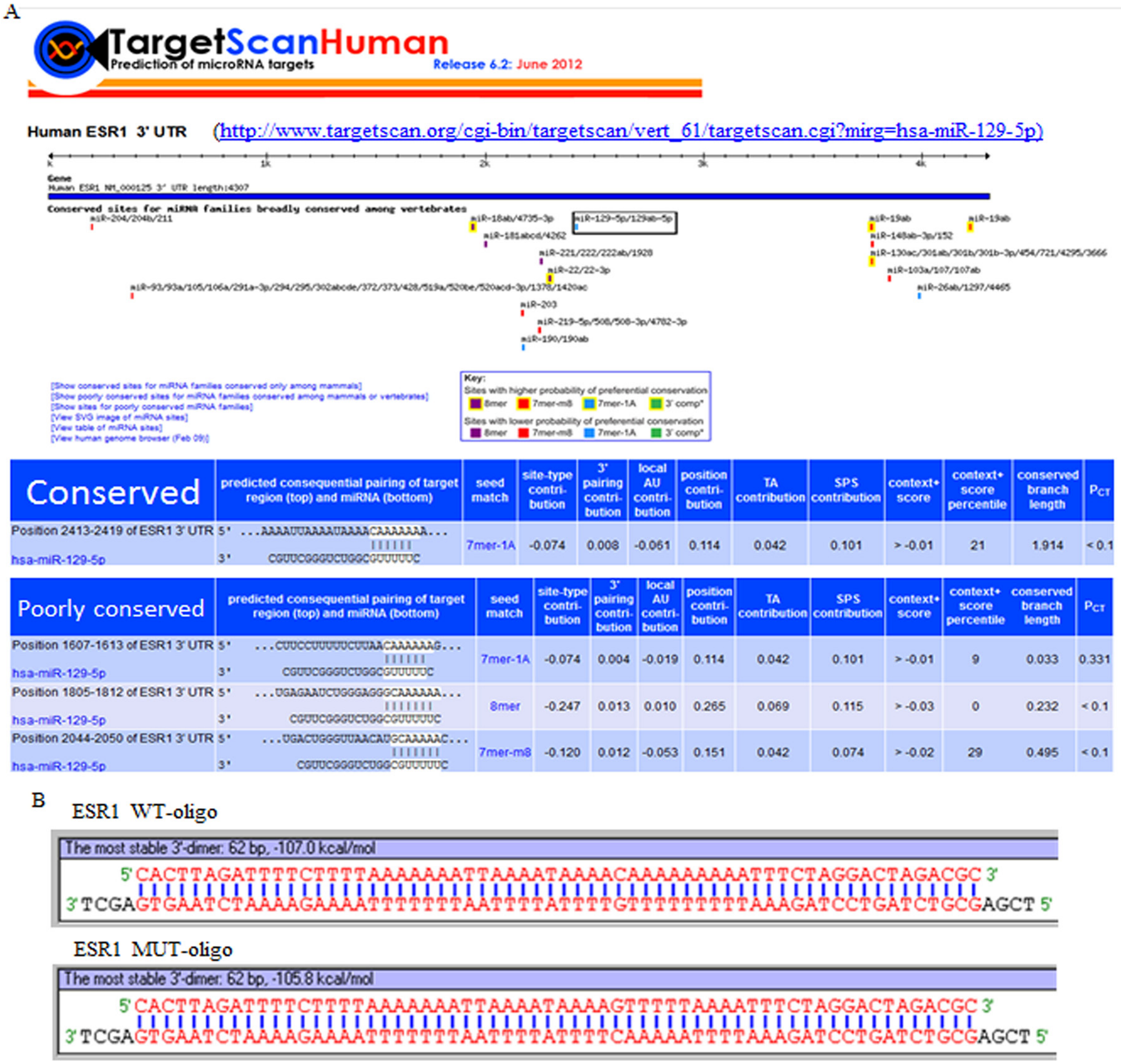

	Sense	Antisense
has-miR-129	CUUUUUGCGGUCUGGGCUUGC	AAGCCCAGACCGCAAAAAGUU
Mimics NC	5' UUCUCCGAACGUGUCACGUTT 3'	5' ACGUGACACGUUCGGAGAATT 3'

Supplementary Figure 1: Informatic analysis of the binding site between miR-129 and ESR1. (A) Potential binding sites between miR-129 and the 3'UTR of ESR1 was detected after analyzing and searching results of DIANA-MICROT, MIRDB, MICRORNA. ORG, TARGETSCAN-VERT, TARGETSCAN-VERT online, and the potential results of bioinformatics analysis from "Target Scan" was recorded. **(B)** Plasmid with wild-typed 3'UTR or mutant 3'UTR, which were introduced into 293T cells.

Supplementary Table 1: Clinicopathologic characteristics of patients

Characteristic	Number (%)
**Total**	42(100%)
**Age(years)**	
**<55**	18(43%)
**≥55**	24(57%)
**Gender**	
**male**	0(0%)
**female**	42(100%)
**Tumor size(cm)**	
**≤ 3**	20(48%)
**>3**	22(52%)
**TNM tumor stage**	
**I+II**	28(67%)
**III+IV**	14(33%)
**Degree of differentiation**	
**Well and moderately**	30(71%)
**Poorly**	12(29%)
**Lymph node metastasis**	
**Yes**	6 (14%)
**No**	36(86%)
**Neoadjuvant chemotherapy**	
**Yes**	15(36%)
**No**	27(64%)
